# Comparing DNA quantity and quality using saliva collection following food and beverage consumption

**DOI:** 10.1186/s13104-019-4211-6

**Published:** 2019-03-23

**Authors:** Summer R. Hughes, Richard R. Chapleau

**Affiliations:** 1 1st American Systems and Services, Falls Church, VA USA; 2 Applied Technology & Genomics Division, Aeromedical Research Department, U.S. Air Force School of Aerospace Medicine, 711th Human Performance Wing, Air Force Research Laboratory, 2510 Fifth St, Dayton, Wright-Patterson AFB, OH 45433 USA

**Keywords:** Saliva sampling, DNA collection methods, Non-invasive sampling, Genetic methods

## Abstract

**Objective:**

With the democratization of genetic testing, researchers, clinicians, and educators must consider the varying degree of field conditions when collecting samples for genetic analyses. For genotyping or sequencing studies, study designers have multiple options from which to choose, including cheek swabs and saliva sampling. One significant benefit of saliva collection is that it can be done remotely, in the privacy of one’s home. This same benefit adds a risk of compliance. Therefore, our goal with this study was to see if the quality and quantity of the saliva collection by a saliva DNA collection kit would be affected by not following the manufacturer’s directions, i.e., drinking or eating right before collection.

**Results:**

We asked five participants to collect saliva samples according to the manufacturer’s guidance and also after consuming five food items or beverages. We evaluated DNA quantity and quality post-purification using spectroscopy, electrophoresis, and polymerase chain reaction genotyping. Consistent with our hypothesis, we did not see a difference in quantity or quality of the isolated DNA. From our results, we conclude that the manufacturer’s instructions serve as an ideal guideline, but the collection devices are robust enough to permit flexibility in sampling at home or in the field.

**Electronic supplementary material:**

The online version of this article (10.1186/s13104-019-4211-6) contains supplementary material, which is available to authorized users.

## Introduction

Since the original publication of the first drafts of the human genome in the early 2000s, the genetics research field has exploded in public popularity. It is now commonplace for the public to use the terms “genetics” and “precision medicine.” In fact, some direct-to-consumer companies are even corporate sponsors for public radio and advertise during commercial breaks of sporting events. The relative ease with which the public now has access to the highly advanced genetic technologies raises a question: how robust are sample collection methods and systems?

Most direct-to-consumer and educational collection systems now rely upon saliva collection methods, either via swabs, oral rinses, or spit tubes. The results of multiple studies show that these collection methods can be rich in DNA quantity with high enough quality to be useful in the research and non-clinical setting [1–4]. More recently, a head-to-head comparison with the gold-standard of DNA sample collection methods, blood sampling, demonstrated that saliva is an acceptable alternative [5]. Unlike blood, which needs to be processed as soon as physically possible, saliva samples require no pre-processing and are commonly collected in methods that promote stability at room temperature for weeks to months [6].

A critical concern with any self-administered test is compliance with the printed manufacturer’s guidance. For saliva collection devices, one of the salient points is to avoid food and beverage consumption for at least one-half hour prior to collecting the sample [7]. In private, educational, or time-restricted settings, this half hour is often not possible. Working adults often have compressed evening schedules including dinner and social engagements, schools have snack and recess breaks scattered throughout the day, and health clinics have a demand for high patient throughput. The ability to obtain adequate samples without collection method restrictions would enhance the clinic’s ability to improve operations and clinical encounter resolution. Therefore, the goal of our study was to test the robustness of a saliva sampling collection tube after consumption of various foods and beverages and compare the results against the gold-standard non-invasive method of a cheek swab. From prior anecdotal observations in our lab from field sampling during athletic events, we hypothesized that beverage consumption would not negatively impact DNA quality or quantity.

## Main text

### Materials and methods

We obtained approval from the Air Force Research Laboratory’s human subjects protection institutional review board, protocol number FWR20180101H. Subjects were briefed individually and signed consent documents witnessed by a third party. Samples were collected from five subjects using the Epicentre Catch-All™ Sample Collection Swab (Epicentre, Madison, WI) and the Isohelix GeneFiX™ Saliva DNA Collection Kit (Isohelix, Kent, UK). All collected samples were collected on the same day with at least 30 min between each collection or over the course of 3 days, due to participant timing constraints. Two swabs were collected per subject; each swab was rubbed against the inside of each cheek for 10–15 s. Six spit samples were collected: per manufacturer’s instructions, immediately after taking a drink of water, eating lunch, drinking a carbonated soft drink, drinking a sports drink, or drinking coffee.

The swabs were extracted using the Epicentre QuickExtract™ DNA Extraction Solution 1.0 (Epicentre). The first swab was processed as is and the second was purified using the Wizard™ SV Genomic DNA Purification System (Promega, Madison, WI) using the microcentrifuge protocol for the purification of genomic DNA from tissue culture cell lysates. There was no cell wash step because the samples were already in solution. The spit samples were extracted using the QIAamp Blood DNA Mini Kit (Qiagen, Valencia, CA) with the manufacturer’s modified saliva extraction protocol.

Samples were quantified using the NanoDrop 1000 (Thermo Fisher Scientific, Waltham, MA) and the Qubit 4.0 (Thermo Fisher Scientific) with the Qubit dsDNA High Sensitivity Assay Kit. The NanoDrop was also used to assess the purity of the samples by comparing the absorbance values at 260 and 280 nm (A260/A280). Both were used per manufacturer’s protocols. Extracted samples, normalized to 10 ng/μL based on Qubit concentrations, were analyzed using a 2% E-Gel^®^ Precast Agarose Gel (Thermo Fisher Scientific). The Agilent Bioanalyzer (Agilent, Santa Clara, CA) along with the High Sensitivity DNA Chip was used to determine the quality of the extracted DNA.

The samples were then analyzed on the Applied Biosystems™ 7500 Fast Real-Time PCR System (Applied Biosystems, Foster City, CA) to test for two genetic markers linked to peanut allergies: rs7192 and rs9275596 [1]. Genotyping with fast ramp pre-polymerase chain reaction (PCR) setting of 60 °C for 1 min, a hold cycle of 95 °C for 10 min, 40 cycles of 95 °C for 15 s then 60 °C for 1 min, and a post-PCR at 60 °C for 1 min. TaqPath ProAmp Master Mix (Thermo Fisher Scientific) was used. The single nucleotide polymorphism (SNP) assays were used at half the manufacturer recommended concentration. The final volume was 10 µL.

### Results

The swab extraction method using the QuickExtract solution resulted in a high quantity of DNA. The values of DNA obtained from the swab without any further processing ranged from 1.38 to 54.2 pg/µL, as determined by the Bioanalyzer (Additional file 1: Table S1). However, previous unreported results from our lab demonstrated that sequencing experiments were inhibited when libraries were prepared directly from the QuickExtract solution extractions. Therefore, we purified these samples using a Wizard column purification protocol, decreasing the concentration, as expected (ranging from 2.59 to 38.2 pg/µL), and improving the A260/A280 ratios to closer to the generally accepted range (1.8–2.1).

**Fig. 1:**
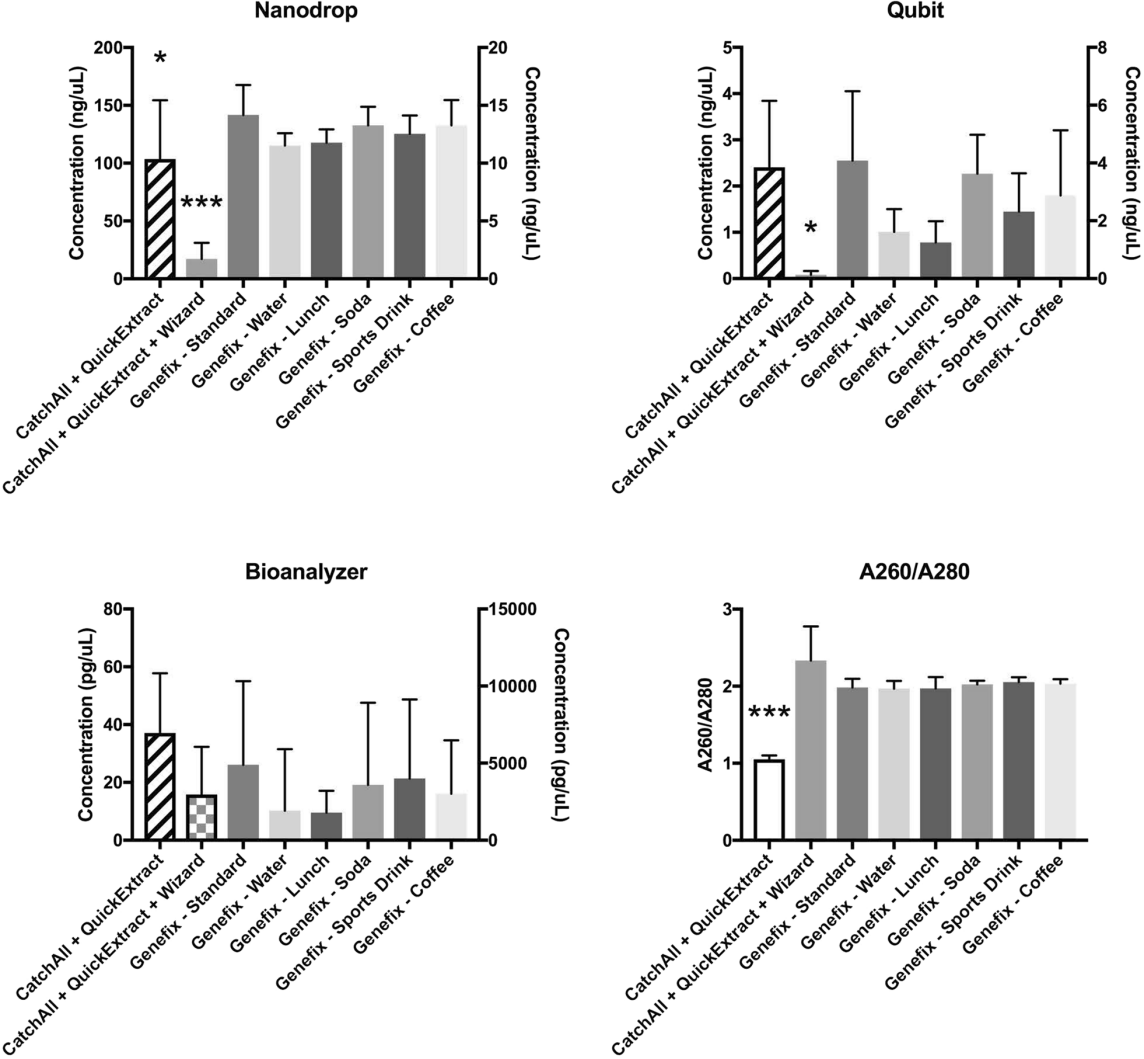
Sample concentrations by three different methods of quantification (Nanodrop, Qubit, and Bioanalyzer) and a purity assessment by the ratio of absorbance at 260 and 280 nm. “CatchAll” conditions are the two swab collections and “Genefix” are the six spit collections. Swab sample concentrations are plotted on the left y-axis while spit sample concentrations are plotted on the right y-axis. Two axes were used as the swab sample without secondary purification (CatchAll + QuickExtract) contains contaminants that absorb at 260 nm in the Nanodrop. *p < 0.05, ***p < 0.001

Spit samples collected per manufacturer’s protocol and extracted using the QIAamp Blood DNA Kit had a range of 1.01 ng/μL to 8.20 ng/μL. The purity indicated by the A260/A280 measurement was typically in range for DNA (see Additional file 1: Table S1). None of the spit samples collected after the subjects had drunk various drinks or had lunch showed DNA concentrations and purity readings significantly different from those collected using the manufacturer’s protocol (30 min of fasting) (Fig. 1).

Extracted samples normalized to 10 ng/μL as determined by Qubit 4.0 readings were run on a 2% E-Gel using a 1-kb ladder (Additional file 1: Figure S1). We expected to see a band toward the top of the gel representing a high molecular weight DNA extraction. As expected, the extracted DNA from the various saliva collection methods was of high molecular weight. Despite being normalized to a 10-ng/µL input, most of the swab samples did not result in a clear band, suggesting either the presence of charge interferents or some other systematic issue.

**Fig. 2:**
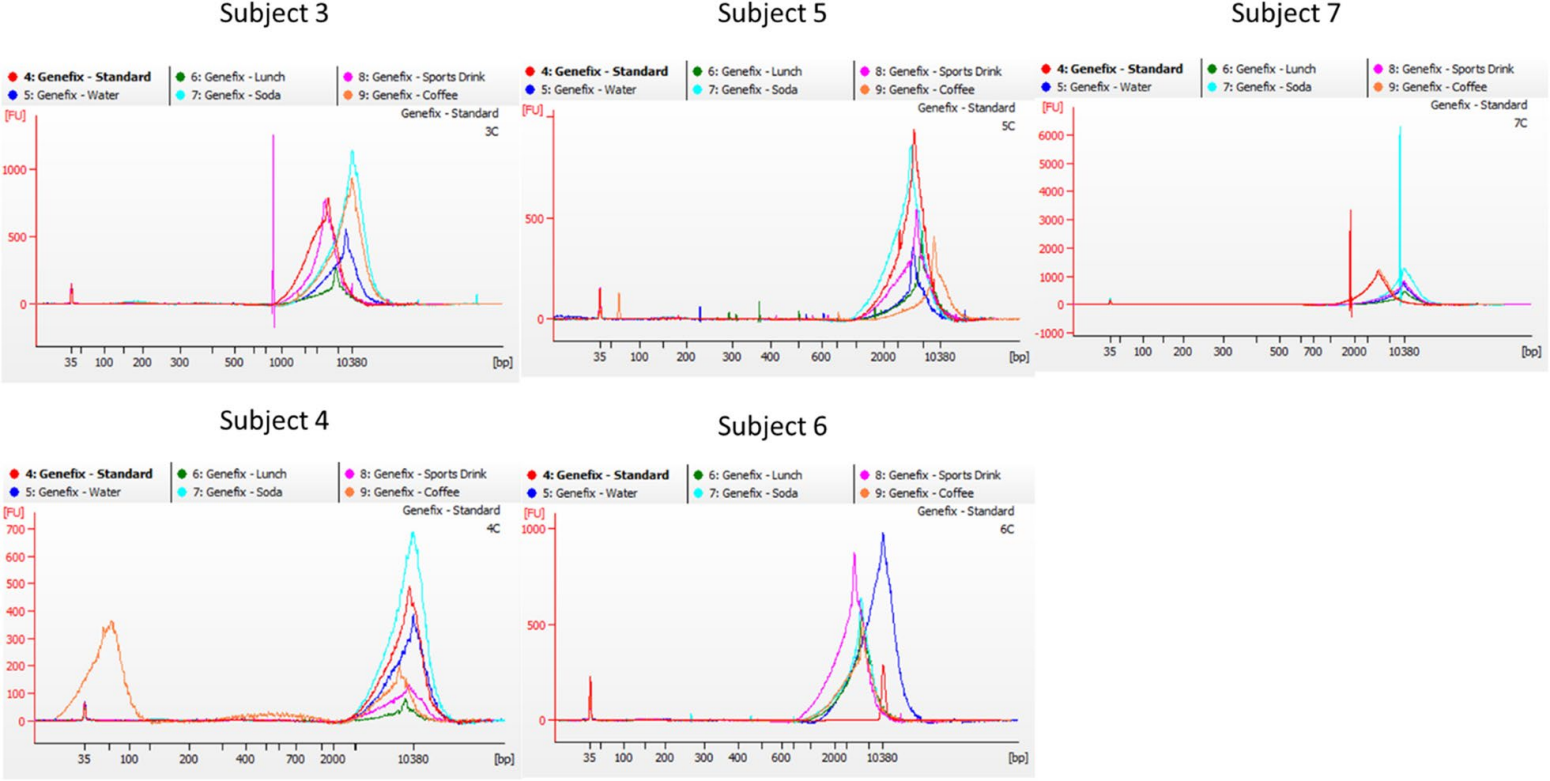
Bioanalyzer electropherogram traces of the spit samples. Each subject is shown individually with colors representing collection conditions: following manufacturer’s instructions (red), after consuming water (blue), after eating lunch (green), after consuming a carbonated beverage (cyan), after consuming a sports drink (magenta), and after consuming coffee (orange)

More detailed size and purity analysis on a Bioanalyzer showed all samples and all conditions to have predominantly high molecular weight DNA and similar concentrations (Fig. 2). Other than the aforementioned low concentration following purification of the swab extraction, there are no collection condition (eating or drinking) related trends in DNA quality, size, or concentration that become apparent when analyzing the electropherogram signals. There does seem to be a subject-dependent change, although this phenomenon was not pursued further.

**Fig. 3:**
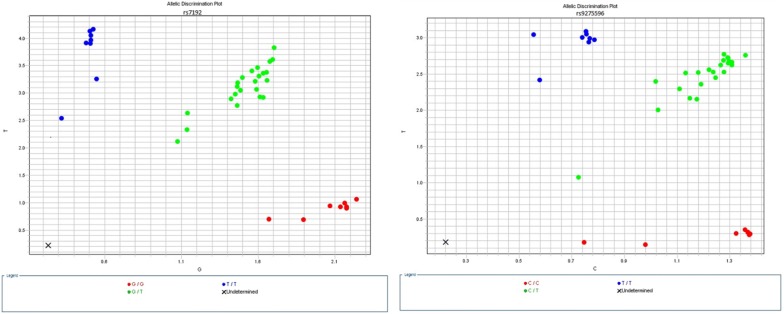
Allelic discrimination plots for rs7192 and rs9275596. The “X” indicates a sample that did not amplify (Subject 5B). Red/bottom samples are homozygous for allele 1 (X-axis), green/central samples are heterozygous, and blue/left samples are homozygous for allele 2 (Y-axis)

Finally, we used SNP genotyping to test the suitability of the extracted DNA for downstream molecular processes (Fig. 3). All of the extracted samples from all subjects were successfully able to produce usable results for genotyping of two SNPs associated with peanut allergies [8], with the exception of one of the purified swab samples (Subject 5B).

### Discussion

When designing a genetic research study, commercial product, or healthcare intervention, usability and end-user compliance are two critical considerations. Blood collection devices that require skin pricks may have a lower compliance due to aversion to pain. From observations in our lab, we have anecdotally noticed a decreased enrollment rate in studies when the primary difference is venipuncture vs. saliva sampling. We have not performed a controlled study to investigate this phenomenon; rather, we have elected to proceed with saliva sampling based on ease of use, apparent increased in enrollment, and the type of research studies predominantly performed in our lab. As our studies involve self-administration of the saliva sampling, we sought to determine if there is a high enough quantity and quality of DNA present for use in downstream molecular applications following various likely field collection conditions, primarily different food and beverage intake. The study design was not intended for cross-comparison between individuals; rather, we sought to compare intra-subject variability of sample collection. With this goal in mind, we observed no significant decrease in DNA quantities caused by variation from the manufacturer’s instructions to fast for 30 min prior to collection.

Although quantity of DNA is important, more critical for advanced molecular biology techniques is the quality of DNA obtained. In our study, we found that collecting saliva samples after eating and drinking did not impact the yield of high molecular weight DNA. Such DNA is critical for next generation sequencing, array scanning, and PCR genotyping. Furthermore, an experimental test using two independent genetic targets in PCR genotyping demonstrated that these samples were amenable to molecular analyses.

Our intended goal with this study was to see if the quality and quantity of the DNA from the saliva collection by a saliva DNA collection kit would be affected by not following the manufacturer’s directions, i.e., drinking or eating right before collection. Consistent with our hypothesis, we did not see a difference in quantity or quality of the isolated DNA.

### Conclusion

Genetic studies have extended beyond the realm of purely a scientific endeavor and are now common among the consumer public. To optimize human samples for these studies, manufacturers are striving to develop simple, robust collection methods. Saliva sampling is the least invasive of these methods, yields high quality DNA, and provides excellent product stability. Our study of the DNA obtained from saliva following consumption of various drinks and/or snacks has shown that there is no significant difference in the quantity and quality of DNA collected using the Isohelix GeneFiX Saliva DNA Collection Kit (Isohelix) when samples are not collected per manufacturer’s protocol. Even though the sample size in this study is small, observations from other studies in our laboratory are consistent with the results of our systematic comparison presented here. We therefore conclude that saliva collection is a robust, non-invasive way to gather samples both in the laboratory and in the field, and the 30-min requirement to abstain from food and beverage is an ideal and not an absolute.

## Limitations

This study uses a small sampling of a single saliva collection device and a single purification kit. Other collection devices and purification kits may require optimization within individual laboratories and may vary in performance. The purified DNA collected in this study was not used for sequencing or microarray genotyping, which are advanced molecular techniques highly sensitive to DNA quality. We expect that the products resulting from alternative collection or purification will still be useful in downstream molecular assays and that those collected using the methods reported here will be useful in advanced genetic experiments.

## Additional file

**Additional file 1: Figure S1.** Gel electrophoresis images. **Table S1.** Analytical data from collections. Image of the gel electrophoresis experiment and raw data collected for all samples and procedures.

## Abbreviations

A260/A280: ratio of absorbance values at 260 and 280 nm
DNA: deoxyribonucleic acid
dsDNA: double-stranded deoxyribonucleic acid
PCR: polymerase chain reaction
SNP: single nucleotide polymorphism
