# Supplementary material for: Comparing DNA quantity and quality using saliva collection following food and beverage consumption
Source: BMC Res Notes. 2019 Mar 23;12:165. doi: 10.1186/s13104-019-4211-6 (PMC6431066; doi:10.1186/s13104-019-4211-6)
Supplement: Supplementary file 1 — Additional file 1: Figure S1. Gel electrophoresis images. Table S1. Analytical data from collections. Image of the gel electrophoresis experiment and raw data collected for all samples and procedures. [file 13104_2019_4211_MOESM1_ESM.docx]

**Additional file for “Comparing DNA quantity and quality using saliva collection following food and beverage consumption”**

Summer R Hughes^1,2^ and Richard R Chapleau^2^*

^1^1^st^ American Systems and Services, Falls Church, VA, USA; ^2^Applied Technology & Genomics Division, Aeromedical Research Department, U.S. Air Force School of Aerospace Medicine, 711th Human Performance Wing, Air Force Research Laboratory, Wright-Patterson AFB, OH, USA

*Correspondence: RRC, richard.chapleau.1@us.af.mil; p: 937-938-2971; 2510 Fifth St, Wright Patterson AFB, OH, USA, 45433


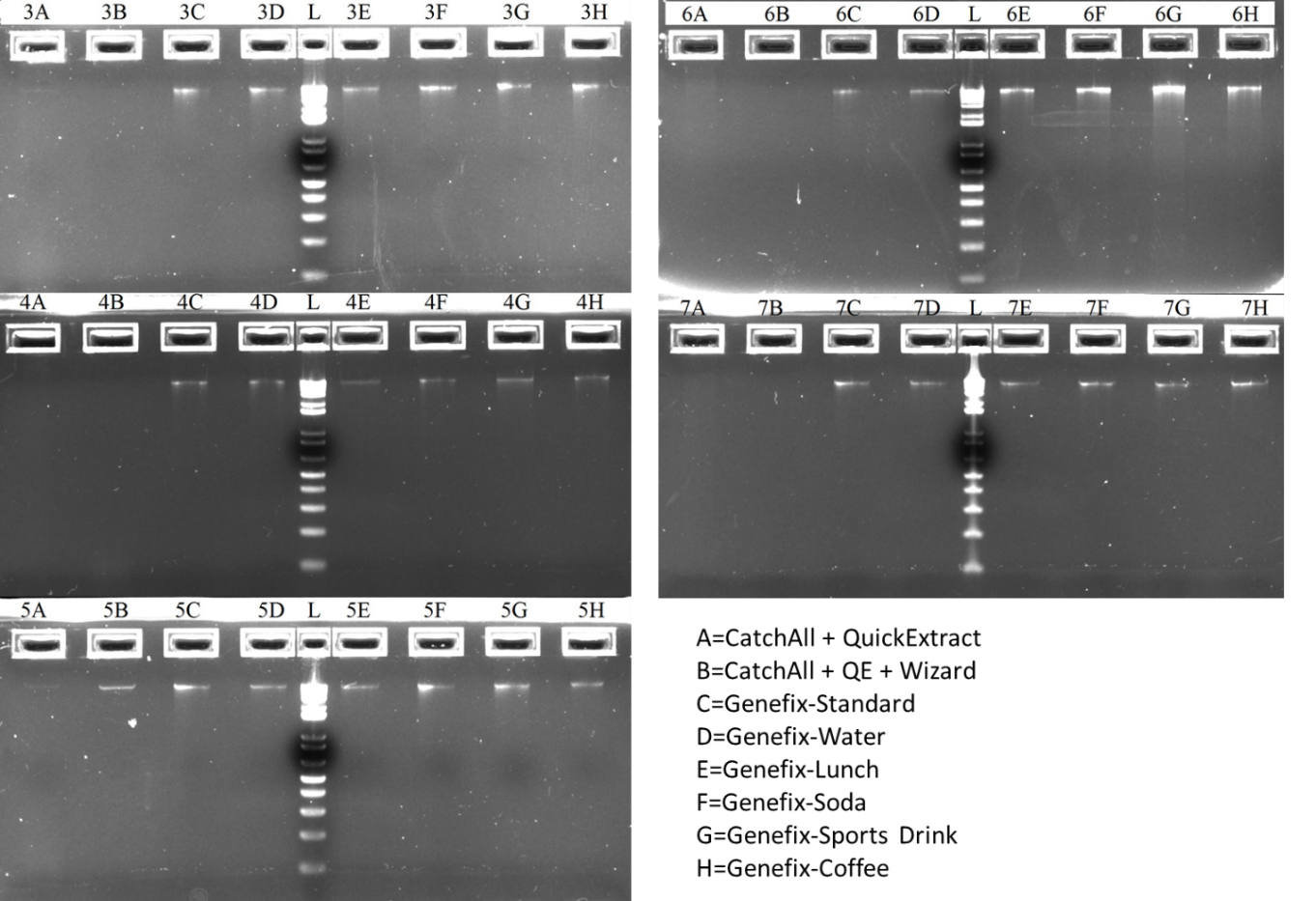


**Figure S1** Gel electrophoresis of extracted samples. Extractions from each subject are shown individually, as described in the legend. All samples were normalized to 10 ng/µL input concentration.

**Table S1: Analytical data from collections**

| **Collection Method** | **Sample** | **Nandrop (ng/uL)** | **260/280** | **Qubit (ng/uL)** | **Bioanalyzer (pg/uL)** | **Molarity (pmol/L)** |
| --- | --- | --- | --- | --- | --- | --- |
| CatchAll + Quick Extract | 3A | 52.8 | 1.01 | 0.99 | 45.95 | 64.2 |
|  | 4A | 149.6 | 1.08 | 3.94 | 43.83 | 7.2 |
|  | 5A | 46.1 | 1.12 | 0.76 | 40.27 | 58.8 |
|  | 6A | 120.7 | 1.04 | 3.32 | 1.38 | 0.5 |
|  | 7A | 148.8 | 1 | 3.02 | 54.21 | 72.3 |
| CatchAll + QE + Wizard | 3B | 1.2 | 2.23 | 0.04 | 2.59 | 3.2 |
|  | 4B | 4.2 | 2.32 | 0.24 | 38.15 | 6.4 |
|  | 5B | 1.1 | 2.7 | 0.31 | 28.74 | 7.5 |
|  | 6B | 1 | 2.76 | 0.03 | 4.5 | 6.6 |
|  | 7B | 1.1 | 1.66 | 0.02 | 5.14 | 3 |
| GeneFiX-Standard | 3C | 13.3 | 2.1 | 3.6 | 13276.47 | 6226.4 |
|  | 4C | 13.5 | 1.87 | 2 | 174.57 | 28.8 |
|  | 5C | 12.2 | 1.89 | 2.88 | 6163.49 | 2224.2 |
|  | 6C | 18.7 | 2.1 | 8.2 | 1.55 | 1.7 |
|  | 7C | 13.2 | 1.96 | 3.74 | 4864.54 | 2077 |
| GeneFiX-Water | 3D | 11.4 | 1.95 | 2.44 | 324.95 | 90.9 |
|  | 4D | 13.1 | 1.87 | 1.79 | 94.42 | 15.4 |
|  | 5D | 10.7 | 1.95 | 0.81 | 9062.16 | 3033.7 |
|  | 6D | 10.4 | 2.14 | 0.77 | 34.61 | 13.2 |
|  | 7D | 11.9 | 1.93 | 2.26 | 48.6 | 11.8 |
| GeneFiX-Lunch | 3E | 11 | 2.14 | 0.92 | 2183.98 | 750.7 |
|  | 4E | 13.4 | 1.83 | 0.21 | 811.77 | 132.3 |
|  | 5E | 12.5 | 1.87 | 1.55 | 2230.37 | 651.9 |
|  | 6E | 11.4 | 2.12 | 2.16 | 3699.24 | 1338.2 |
|  | 7E | 10.6 | 1.9 | 1.41 | 50.8 | 12.4 |
| GeneFiX-Soda | 3F | 15.9 | 2.03 | 5.92 | 26.87 | 10.6 |
|  | 4F | 13.3 | 2.02 | 3.24 | 137.15 | 22.5 |
|  | 5F | 11.6 | 2.08 | 2.84 | 12128.08 | 5265.5 |
|  | 6F | 12.9 | 2.04 | 2.52 | 5561.85 | 2196.6 |
|  | 7F | 12.7 | 1.95 | 3.64 | 87.51 | 19.4 |
| GeneFiX-Sports Drink | 3G | 12.2 | 2.09 | 2.72 | 2216.4 | 2216.4 |
|  | 4G | 11.9 | 2.04 | 0.62 | 221.89 | 37.5 |
|  | 5G | 10.7 | 2.02 | 1.36 | 5076.22 | 1875.2 |
|  | 6G | 15 | 2.14 | 3.94 | 12436.97 | 6470 |
|  | 7G | 12.9 | 1.98 | 2.96 | 69.88 | 18.8 |
| GeneFiX-Coffee | 3H | 16.8 | 2.04 | 6.22 | 105.43 | 31.5 |
|  | 4H | 13.6 | 1.97 | 1.09 | 1401.81 | 231.1 |
|  | 5H | 11.1 | 2.08 | 0.83 | 207.77 | 46.1 |
|  | 6H | 12 | 1.98 | 2.26 | 5747.75 | 2366.9 |
|  | 7H | 12.9 | 2.1 | 4 | 7642.72 | 2878.9 |
